# Cervical lymph node response to 131I therapy in differentiated thyroid cancer using radiomics and clinical features

**DOI:** 10.3389/fonc.2026.1860423

**Published:** 2026-06-23

**Authors:** Yi Ruan, Hui Yuan, Feng Zheng, Xuehua Chen, Cheng Xu

**Affiliations:** Nuclear Medicine, Changde Hospital, Xiangya School of Medicine, Central South University (The First People’s Hospital of Changde City), Changde, China

**Keywords:** bioinformatics, C-X-C motif chemokine ligand 8, differentiated thyroid cancer, predictive model, radioactive iodine-131 therapy, radiomics

## Abstract

**Background:**

To develop and validate a radiomics-based predictive model integrating clinical and imaging features to evaluate cervical lymph node response to I-131 therapy in differentiated thyroid cancer (DTC).

**Methods:**

A retrospective cohort of patients with pathologically confirmed DTC who underwent cervical lymph node dissection followed by 131I therapy was analyzed. Patients were stratified into Excellent and Non-Excellent response groups according to biochemical, imaging, and follow-up outcomes. Key inflammatory-related targets were initially identified through analysis of the GSE33630 dataset, and representative radiomics features potentially associated with inflammatory activity were subsequently extracted from pre-therapeutic CT images. Feature selection was performed using the least absolute shrinkage and selection operator (LASSO) and multivariable logistic regression to construct predictive models. Model discrimination, calibration, and clinical utility were assessed using AUC, calibration curves, Hosmer–Lemeshow test, and decision curve analysis. A nomogram was further developed to facilitate clinical interpretation.

**Results:**

The integrated clinical–radiomics model demonstrated favorable discrimination in both the training and validation cohorts, with good calibration and net clinical benefit across clinically relevant threshold probabilities.

**Conclusion:**

The proposed radiomics-assisted model shows promising ability to predict cervical lymph node response to 131I therapy in DTC, providing a potentially useful tool to support individualized postoperative management. Nevertheless, external validation and comparison with existing predictive strategies are required before routine clinical adoption.

## 1 Introduction

Differentiated thyroid cancer (DTC) accounts for 80%–90% of all thyroid malignancies and generally has a favorable prognosis; however, approximately 20% of patients develop postoperative cervical lymph node metastases (LNM) or local recurrence (1, 2). Radioactive iodine-131 (131I) therapy is a critical approach for eradicating residual and metastatic lesions after surgery, and its efficacy is influenced by multiple factors, including tumor differentiation, metastatic burden, and molecular characteristics (3, 4). Nevertheless, in clinical practice, a subset of patients exhibit suboptimal or poor responses to 131I therapy, presenting with persistent lesions or radioactive iodine-refractory (RAIR) status, which poses considerable challenges for subsequent treatment decision-making and long-term disease management (5, 6).

Currently, prediction of 131I treatment response relies primarily on conventional indicators such as serum thyroglobulin (Tg) levels, pathological staging, and imaging findings (7). However, these markers demonstrate limited sensitivity and specificity and are unable to fully capture the pronounced biological heterogeneity of metastatic lymph nodes. Radiomics, a technique that extracts high-dimensional quantitative descriptors from routine medical images (8), offers the potential to characterize intratumoral heterogeneity, microstructural complexity, and tumor microenvironmental features (9), and has shown increasing value in risk stratification and outcome prediction across multiple malignancies. Nonetheless, in the context of DTC, the relationship between radiomics-derived phenotypes and iodine-131 therapeutic response has not been sufficiently elucidated, and systematic biological interpretation of radiomics signatures remains limited.

Recent studies have highlighted that inflammatory and chemotactic signaling pathways play pivotal roles in thyroid cancer progression, tumor microenvironment remodeling, and alterations in iodine uptake function (10–12). Chemokines such as C-X-C motif chemokine ligand 8 (CXCL8/IL-8) may modulate treatment sensitivity through promoting angiogenesis, immune-inflammatory activation, and metastatic niche adaptation, thereby potentially contributing to heterogeneous 131I responses (13–15). Integrating molecular bioinformatics analysis with imaging phenotypes may therefore help clarify the “imaging–molecular coupling” mechanisms underlying different therapeutic outcomes and enhance the biological interpretability of radiomics-based prediction.

Recent advances in artificial intelligence (AI) and machine learning have substantially expanded the application of imaging-based prediction models in thyroid cancer. A recent systematic review and meta-analysis published in 2025, including 35 studies, reported a pooled AUC of 0.818 (95% CI: 0.788–0.848) for AI-based prediction models of thyroid cancer metastasis (16). Although these findings demonstrate the potential utility of AI-assisted risk stratification, considerable heterogeneity in study design, imaging protocols, feature selection strategies, and validation approaches was observed across studies. These limitations highlight the need for more robust, interpretable, and clinically translatable predictive models that integrate biological and imaging information (17). Common pitfalls such as model overfitting, inadequate sample size justification, lack of calibration and clinical utility assessment, and limited external generalizability have been increasingly recognized in recent literature (18, 19). These challenges underscore the need for rigorously designed and clinically meaningful predictive models, rather than purely technical explorations.

Based on these considerations, this study integrates multidimensional clinical information, radiomics features, and transcriptomic data to construct a multimodal predictive model with the following aims (1): to identify key clinical and radiomics determinants associated with 131I therapeutic response in cervical LNM of DTC; (2) to explore the potential involvement of chemotaxis-related inflammatory pathways in response variability; and (3) to develop an interpretable, biologically supported, and clinically applicable comprehensive predictive model to support precision stratification and individualized management in DTC.

## 2 Materials and methods

### 2.1 Study design and patient population

This single-center retrospective cohort study included patients with DTC who received radioactive 131I therapy at the Department of Nuclear Medicine, Changde Hospital, Xiangya School of Medicine, Central South University (The First People’s Hospital of Changde City), between January 2019 and January 2024 (20). All patients presented with postoperative cervical LNM confirmed as papillary thyroid carcinoma metastases by ultrasound-guided fine-needle aspiration (FNA) cytology and Tg measurement in needle washout fluid. The overall study workflow, including patient selection, preprocessing, feature selection, and model validation.

Inclusion criteria: (1) Postoperative pathological diagnosis of DTC with suspicious cervical LNM; (2) FNA cytology and Tg washout positive; (3) Availability of contrast-enhanced computed tomography (CT) or magnetic resonance imaging (MRI) scans within one month prior to enrollment; (4) Complete 131I treatment and follow-up data.

Exclusion criteria: (1) Coexistence of other malignancies; (2) Inadequate or missing imaging data; (3) Incomplete follow-up records.

### 2.2 Treatment protocol and response evaluation

All patients received standard 131I therapy and thyroid-stimulating hormone (TSH) suppression therapy. Treatment response was evaluated 6–12 months after therapy according to the 2025 American Thyroid Association (ATA) dynamic risk stratification criteria (20).

Whole-body scintigraphy (WBS) or single-photon emission computed tomography/computed tomography (SPECT-CT) (21) findings were interpreted as “radioiodine-avid” if focal radioactive uptake corresponding to known or suspected lesions (cervical lymph nodes, thyroid bed, or distant metastases) was observed. Physiological uptake or absence of radioactivity was defined as “radioiodine-nonavid” (22). WBS and SPECT-CT images were independently reviewed by two experienced nuclear medicine physicians. Uptake was evaluated using a combination of qualitative visual assessment and semi-quantitative estimation based on lesion-to-background comparison and anatomic correlation on SPECT-CT fusion images. In cases of discrepancy, consensus was reached through joint review. As SUV quantification is not directly applicable to routine WBS imaging, emphasis was placed on standardized semi-quantitative interpretation rather than PET-based SUV metrics.

The therapeutic endpoint was classified according to ATA response categories. An Excellent response was defined as negative imaging findings with suppressed Tg <0.2 ng/mL or stimulated Tg <1 ng/mL, accompanied by negative or declining anti-thyroglobulin antibody (TgAb) levels. All other responses were classified as non-Excellent. Structural evidence of residual or progressive lesions (e.g., radiologic persistence or progression) was categorized as a structural incomplete response and included in the non-Excellent response group.

### 2.3 Clinical and laboratory parameters

Comprehensive clinical data were collected, including demographic information (age, sex), pathological characteristics (histological subtype, primary lesion type and distribution), and metastasis-related parameters such as cervical lymph node region (central compartment level VI or lateral neck), number of metastatic lymph nodes (≤5 or >5), maximum short-axis diameter (mm), and presence of extranodal extension (ENE).

Laboratory parameters included baseline serum Tg, TgAb, TSH, and thyroid function tests (free triiodothyronine [FT3] and free thyroxine [FT4]). According to pre-treatment TSH suppression status, patients were categorized into a well-suppressed group (TSH 0.1–0.5 mU/L) and a poorly suppressed group (TSH below or above this range). Quantitative Tg in FNA washout was documented to confirm the metastatic origin of the lesions.

Detailed 131I treatment parameters including single and cumulative 131I dose, interval between treatments, and TSH stimulation methods were recorded. During efficacy assessment, post-treatment WBS/SPECT-CT results were reviewed in conjunction with structural imaging (CT, MRI, or ultrasound) for integrated evaluation. Radiologic changes and disease progression were monitored throughout follow-up to assess short-term therapeutic efficacy and progression-free survival (PFS).

### 2.4 Bioinformatics analysis

Public dataset: Differential expression analysis was performed using the Gene Expression Omnibus (GEO) dataset GSE33630 (49 papillary carcinoma tissues vs. 11 adjacent normal tissues) under the criteria of adjusted P value <0.01 and |log_2_ fold change| >2. Gene Ontology (GO) and Kyoto Encyclopedia of Genes and Genomes (KEGG) enrichment analyses were conducted with a focus on chemotaxis- and migration-related pathways (e.g., chemokine-mediated signaling, leukocyte/granulocyte chemotaxis).

Candidate gene set: Based on the intersection of enriched pathways and supporting literature evidence (23), a candidate gene set was defined. C-X-C motif chemokine ligand 8 (CXCL8) was identified as a core gene due to its repeated occurrence in multiple pathways and proteomic validation in the Human Protein Atlas (https://www.proteinatlas.org/).

Feature reduction and Geno-score construction: For genes under “leukocyte chemotaxis” (21 genes) and “regulation of granulocyte chemotaxis” (7 genes), least absolute shrinkage and selection operator (LASSO) regression with 10-fold cross-validation (seed = 2022) was performed. Two Geno-score versions were generated -- Simplified 5-gene model: CXCL8, TNFAIP6, DAPK2, C3AR1, S100A14 (lambda.min solution). Extended 10-gene model: ITGA9, CXADR, CXCL8, SERPINE1, CCR1, TNFAIP6, DAPK2, CCL20, GREM1, S100A14. The Geno-score was calculated as: Geno-score = Σ(γ_j × Gene_j), where Gene_j represents standardized expression and γ_j denotes the LASSO coefficient (Note: The bioinformatics analysis was an exploratory secondary analysis of public databases, providing mechanistic consistency and external validation for the retrospective cohort, but not serving as the primary endpoint). Differential expression analysis applied Benjamini–Hochberg false discovery rate (FDR) correction, and the principal findings remained consistent after adjustment.

The transcriptomic analysis was based on the GSE33630 dataset, which was selected because of its well‐curated annotation, consistent experimental platform, and its extensive prior use in thyroid cancer molecular profiling studies. Although this dataset provides reliable biological insight, we acknowledge that reliance on a single cohort may limit generalizability. Therefore, the bioinformatics results were primarily used to provide biological rationale and hypothesis support rather than to draw definitive molecular conclusions, and future research will incorporate additional GEO datasets and TCGA-THCA cohorts for external molecular validation. The bioinformatics analysis was conducted as an exploratory secondary analysis to provide biological interpretation and mechanistic support for the radiomics findings. Transcriptomic-derived variables were not directly incorporated into the final combined clinical–radiomics prediction model.

### 2.5 Radiomics analysis

Image acquisition and lesion segmentation: Pre-treatment contrast-enhanced CT or MRI scans performed within one month before 131I therapy were collected (24). Metastatic lymph nodes were manually segmented one by one using 3D Slicer (version 5.0.3) to generate three-dimensional volumes of interest (VOIs). A three-dimensional (3D) volumetric segmentation approach was applied, and regions of interest (ROIs) were delineated across all lesion-containing slices rather than relying on a single representative slice. Radiomics features were therefore extracted on a volumetric basis, reflecting the overall spatial heterogeneity of metastatic lymph nodes. Image resampling and intensity normalization were performed prior to feature extraction in accordance with the Image Biomarker Standardisation Initiative (IBSI) recommendations to minimize acquisition-related variability. Cervical lymph node lesions were manually segmented on axial CT images by two radiologists (with 8 and 12 years of experience in head and neck imaging), both blinded to clinical and outcome information. To ensure segmentation reliability, 30 randomly selected cases were independently re-segmented after a 3-week interval to assess intra-observer reproducibility, while cross-observer segmentation was used to assess inter-observer reproducibility. Intraclass correlation coefficients (ICC) were calculated, and radiomics features with ICC ≥ 0.80 were considered robust and retained for subsequent modeling, whereas unstable features were excluded from analysis.

Feature extraction and preprocessing: Radiomics features were extracted using PyRadiomics (version 3.0.1), including shape features, first-order statistics, and multiple texture classes—gray-level co-occurrence matrix (GLCM), gray-level run length matrix (GLRLM), gray-level size zone matrix (GLSZM), neighboring gray tone difference matrix (NGTDM), and gray-level dependence matrix (GLDM). Multi-scale filter transformations, including Laplacian of Gaussian (LoG) and wavelet filters, were also applied. All features were standardized using z-score normalization. To mitigate scanner and acquisition variability across devices and timepoints, batch effects were corrected using the ComBat algorithm. Because image harmonization had been completed during the original radiomics preprocessing workflow, scanner-specific ComBat parameters were not available for retrospective re-estimation. Therefore, the potential influence of harmonization-related information leakage was addressed through strict training-only feature selection procedures and additional cross-validation analyses.

Feature selection and Rad-score construction: Highly correlated features (|r| > 0.9) were removed using Pearson correlation analysis. Key features were then selected via LASSO logistic regression, and a radiomics score (Rad-score) was calculated as: Rad-score = Σ(β_i × Radiomics_i), where β_i denotes the regression coefficient of each retained feature.

In addition, conventional imaging features, including lymph node short-axis diameter, long-to-short axis ratio, cystic change, calcification, enhancement pattern, and capsular invasion, were extracted as reference variables for comparative modeling.

### 2.6 Statistical analysis

After normality testing, continuous variables were expressed as mean ± standard deviation (SD) or median [interquartile range, IQR], while categorical variables were expressed as counts and percentages. Categorical variables were coded using clinically interpretable reference categories, as detailed. Data completeness was assessed before statistical analysis. No missing values were identified in the clinical, laboratory, or radiomics variables included in the final analysis dataset. Therefore, multiple imputation was not required and all analyses were performed using complete-case data. Features were retained based on ICC ≥0.80 and correlation |r| ≤0.9. The primary outcome was ATA response (Excellent vs. non-Excellent). Three logistic regression models were constructed: (1) Clinical model: including age, sex, TSH, Tg/TgAb, and lymph node burden; (2) Radiomics model: based on Rad-score; (3) Combined model: clinical + Rad-score (main model).

Model performance was evaluated using receiver operating characteristic (ROC) analysis to calculate the area under the curve (AUC, 95% confidence interval [CI]), sensitivity, and specificity. Calibration was assessed using the Hosmer–Lemeshow goodness-of-fit test, and Internal validation was additionally performed using 1000 bootstrap resamples to reduce optimism bias and assess the stability of model performance to estimate the concordance index (C-index). To further assess model robustness and reduce potential optimism bias, a stratified 5-fold cross-validation analysis was additionally performed in the training cohort. The training data were randomly partitioned into five approximately equal folds while preserving outcome distribution. Model training and evaluation were repeated across all folds, and the mean area under the receiver operating characteristic curve (AUC) with standard deviation (SD) was calculated.

Clinical utility was evaluated by decision curve analysis (DCA). Positive predictive value (PPV) and negative predictive value (NPV) were estimated based on the reported sensitivity and specificity, assuming the observed prevalence of Non-Excellent response in the study cohort, with Non-Excellent response defined as the positive outcome. All feature selection procedures, including correlation filtering, LASSO-based dimensionality reduction, and multivariable model construction, were performed exclusively in the training cohort. The validation cohort was used only for independent performance evaluation and was not involved in feature selection or model optimization.

All analyses and visualizations were performed using GraphPad Prism 10.0 (GraphPad Software, San Diego, CA, USA), R software version 4.3.2 (R Foundation for Statistical Computing, Vienna, Austria), and SPSS version 26.0 (IBM Corporation, Armonk, NY, USA). A two-tailed P value <0.05 was considered statistically significant.

Statistical comparisons were conducted using standard validation metrics including AUC, sensitivity, specificity, calibration assessment, and decision curve analysis, as appropriate.

### 2.7 Ethical statement

This study was approved by the Ethics Committee of Changde Hospital, Xiangya School of Medicine, Central South University (The First People’s Hospital of Changde City) (Approval No. 2025-080-01) and conducted in accordance with the principles of the Declaration of Helsinki (2013 revision) (25). As this was a retrospective analysis, all patient data were anonymized, and the requirement for informed consent was waived by the committee.

### 2.8 Sample size estimation

Sample size requirements for model development were assessed *a priori* using contemporary recommendations for multivariable prediction models. Following the events-per-variable (EPV) principle and the framework proposed by Riley et al. (26), we assumed an anticipated prevalence of Non-Excellent response of approximately 50% and prespecified 8 candidate predictors. Under these assumptions, the pmsampsize package in R indicated that a minimum of about 150 patients (with at least ~75 outcome events) (27, 28) would be required to achieve adequate model stability and limit optimism in model performance. In our derivation cohort, 175 patients were included, yielding more than 80 Non-Excellent responses, which exceeded this minimum requirement and was considered sufficient for reliable estimation of the model parameters.

## 3 Results

### 3.1 Differential expression and functional enrichment analysis

**Figure 1:**
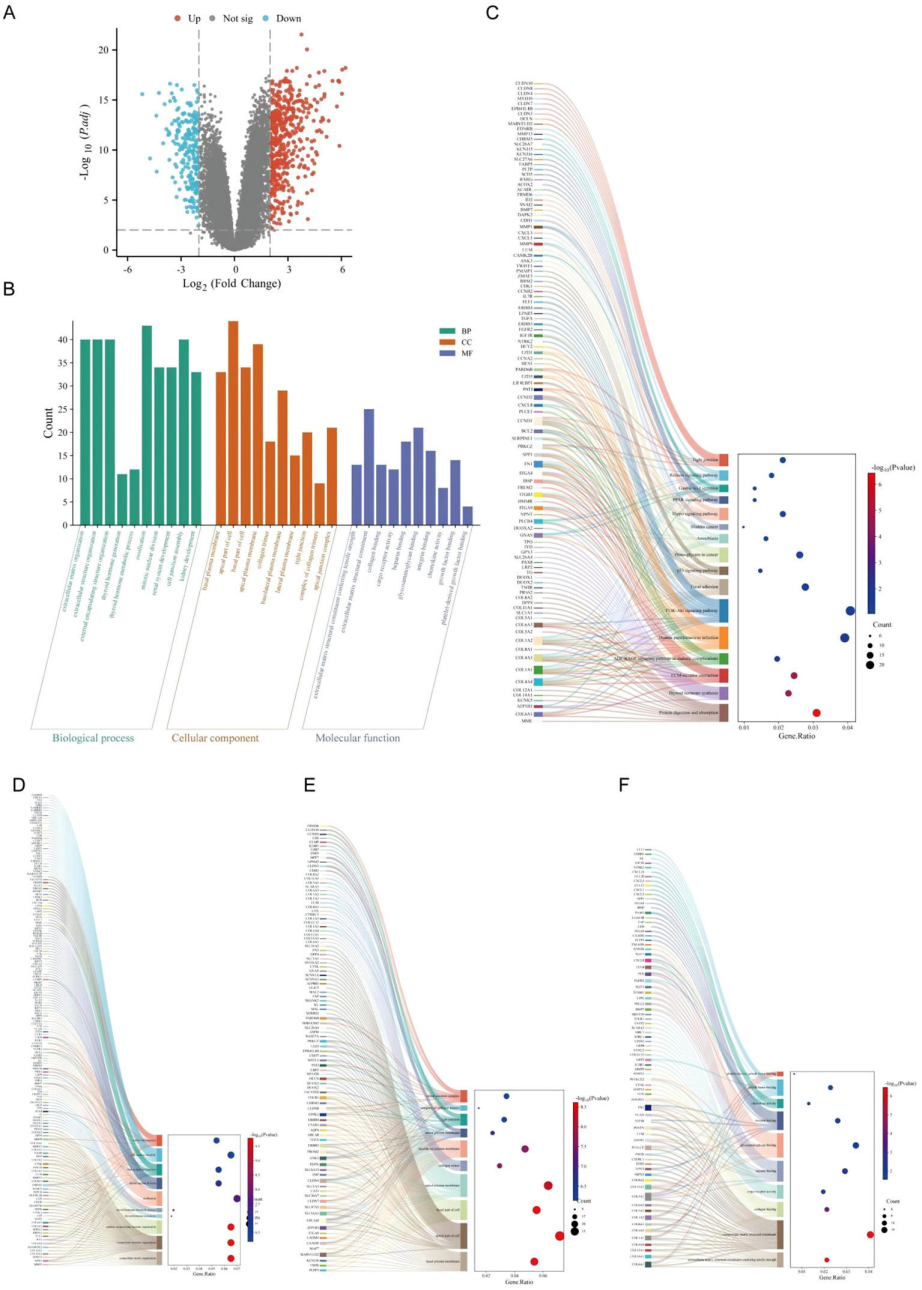
Differential expression and functional enrichment analysis in the GSE33630 dataset. **(A)** Volcano plot displaying differentially expressed genes (threshold: |log_2_FC| > 2, adj.*P*.Val < 0.01); **(B)** Combined GO enrichment bar plot; **(C)** KEGG Sankey diagram showing pathway associations; **(D–F)** Enrichment results for BP, CC, and MF. Key pathways include extracellular matrix remodeling, cell adhesion, chemotaxis, inflammatory response, and the PI3K-AKT signaling axis. GO, Gene Ontology; KEGG, Kyoto Encyclopedia of Genes and Genomes; FC, fold change; BP, biological process; CC, cellular component; MF, molecular function; DEG, differentially expressed gene.

In the GSE33630 dataset (49 PTC vs. 11 adjacent control thyroid [ACT] samples), differentially expressed genes (DEGs) were identified using thresholds of adj.*P*.Val < 0.01 and |log_2_FC| > 2 (**Figure 1A**). GO and KEGG enrichment analyses (**Figures 1B–F**) revealed that these DEGs were primarily enriched in biological processes such as extracellular matrix remodeling, cell adhesion, chemotaxis, inflammatory response, and the PI3K-AKT signaling pathway, suggesting their strong association with tumor migration, invasion, and distant metastasis.

### 3.2 Screening of chemotaxis-related core genes

**Figure 2:**
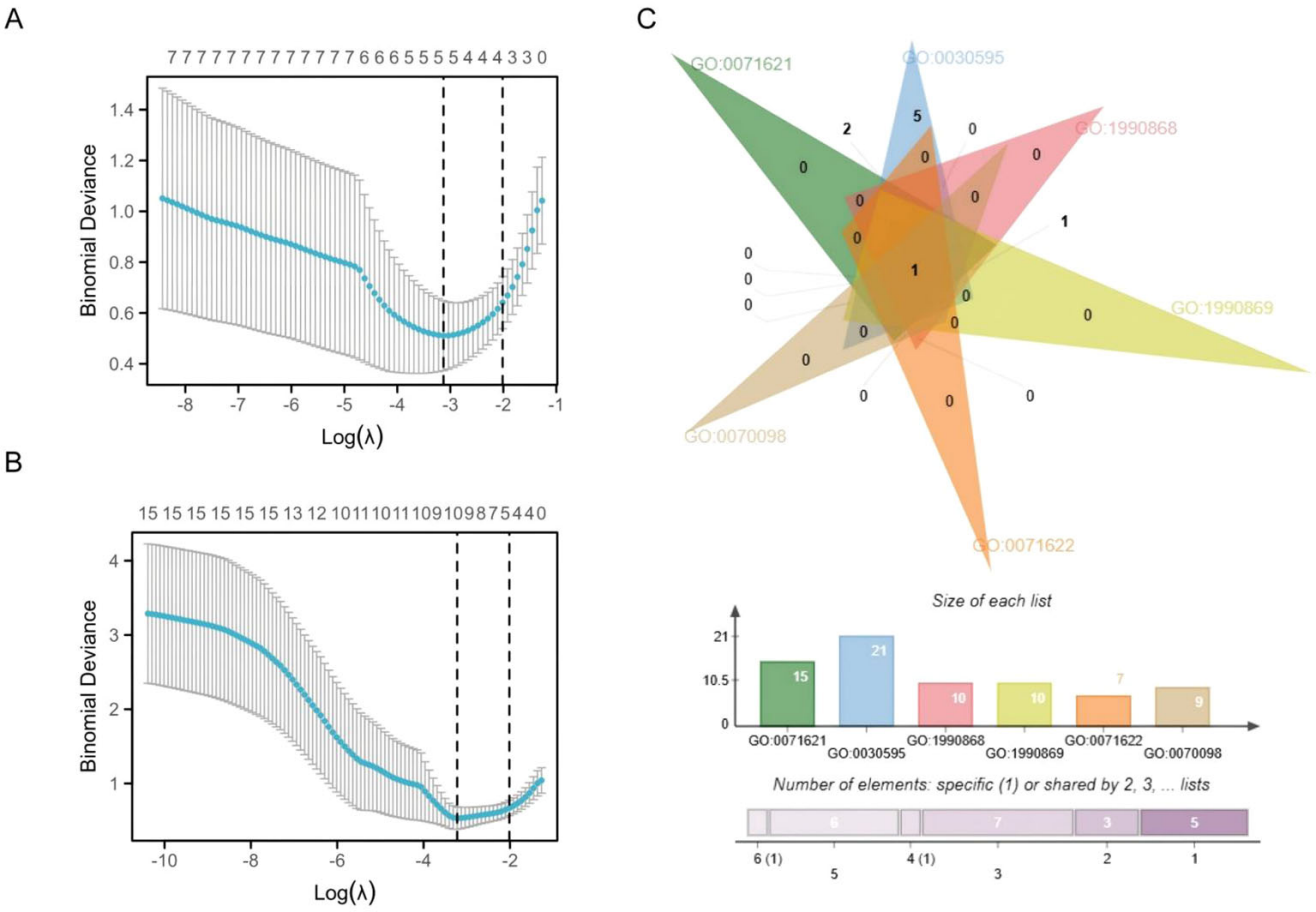
LASSO feature selection and intersection analysis of chemotaxis-related pathways. **(A)** LASSO curve for the regulation of granulocyte chemotaxis pathway; **(B)** Coefficient path plot for the leukocyte chemotaxis pathway; **(C)** Venn diagram illustrating intersections of core chemotaxis and directional migration pathways, with CXCL8 at the central overlap. LASSO, least absolute shrinkage and selection operator.

As shown in **Table 1** and **Figures 2A–C**, 21 candidate genes from chemotaxis-related pathways were included in LASSO regression analysis. At lambda.min = 0.039881, 10 nonzero coefficient genes were identified (ITGA9, CXADR, CXCL8, SERPINE1, CCR1, TNFAIP6, DAPK2, CCL20, GREM1, and S100A14). At lambda.1se = 0.13367, a reduced 5-gene model was obtained. Further LASSO analysis within the “granulocyte chemotaxis” gene set (n = 7) retained CXCL8, TNFAIP6, DAPK2, C3AR1, and S100A14. Notably, CXCL8 was consistently selected in both models, indicating strong feature stability and chemotactic specificity. Venn diagram results confirmed CXCL8 as a central overlapping gene among multiple chemotaxis-related pathways (**Figure 2C**).

**Table 1:** Tenfold cross-validation results of LASSO analysis (chemotaxis-related gene set).

Criterion	lambda	Index	Statistics	SE	Number of nonzero coefficients
Leukocyte chemotaxis					
lambda.min	0.039881	22	0.53343	0.15041	10
lambda.1se	0.13367	9	0.67044	0.10339	5
Regulation of granulocyte chemotaxis					
lambda.min	0.043754	21	0.51067	0.13768	5
lambda.1se	0.13362	9	0.64071	0.10242	3

Statistic = mean cross-validation error; Index = feature dimension index along the penalty path. LASSO, least absolute shrinkage and selection operator; SE, standard error.

### 3.3 Protein-level validation

**Figure 3:**
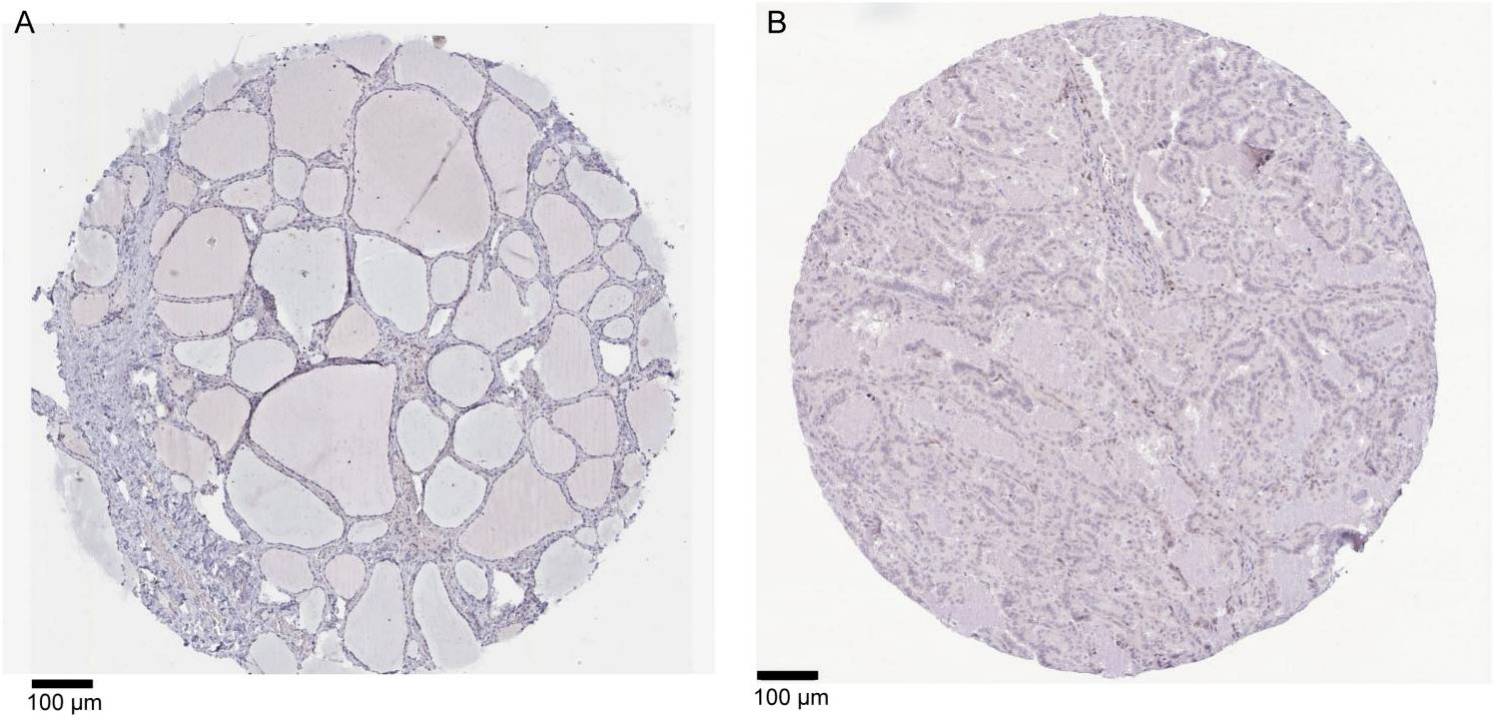
Immunohistochemical expression of CXCL8 in thyroid tissues (HPA database). **(A)** Negative or weak CXCL8 expression in normal thyroid tissue; **(B)** Enhanced CXCL8 staining in papillary thyroid carcinoma tissue, indicating activation of the inflammation–chemotaxis microenvironment. Scale bar = 100 μm. HPA, Human Protein Atlas; CXCL8, C-X-C motif chemokine ligand 8.

Immunohistochemistry data from the HPA database (**Figure 3**) demonstrated high CXCL8 expression in PTC tissues, predominantly localized in follicular epithelial cells, whereas normal thyroid tissues showed low expression. These findings support the involvement of CXCL8 in inflammation- and chemotaxis-driven tumor microenvironment remodeling, consistent with its high recurrence frequency in bioinformatics analysis.

Integrated bioinformatics analysis suggested that CXCL8, together with ITGA9, SERPINE1, TNFAIP6, DAPK2, and S100A14, may represent a potential “chemotaxis-adhesion-inflammation” signaling axis, providing mechanistic insight into therapeutic response heterogeneity.

### 3.4 Baseline characteristics of patients

**Figure 4:**
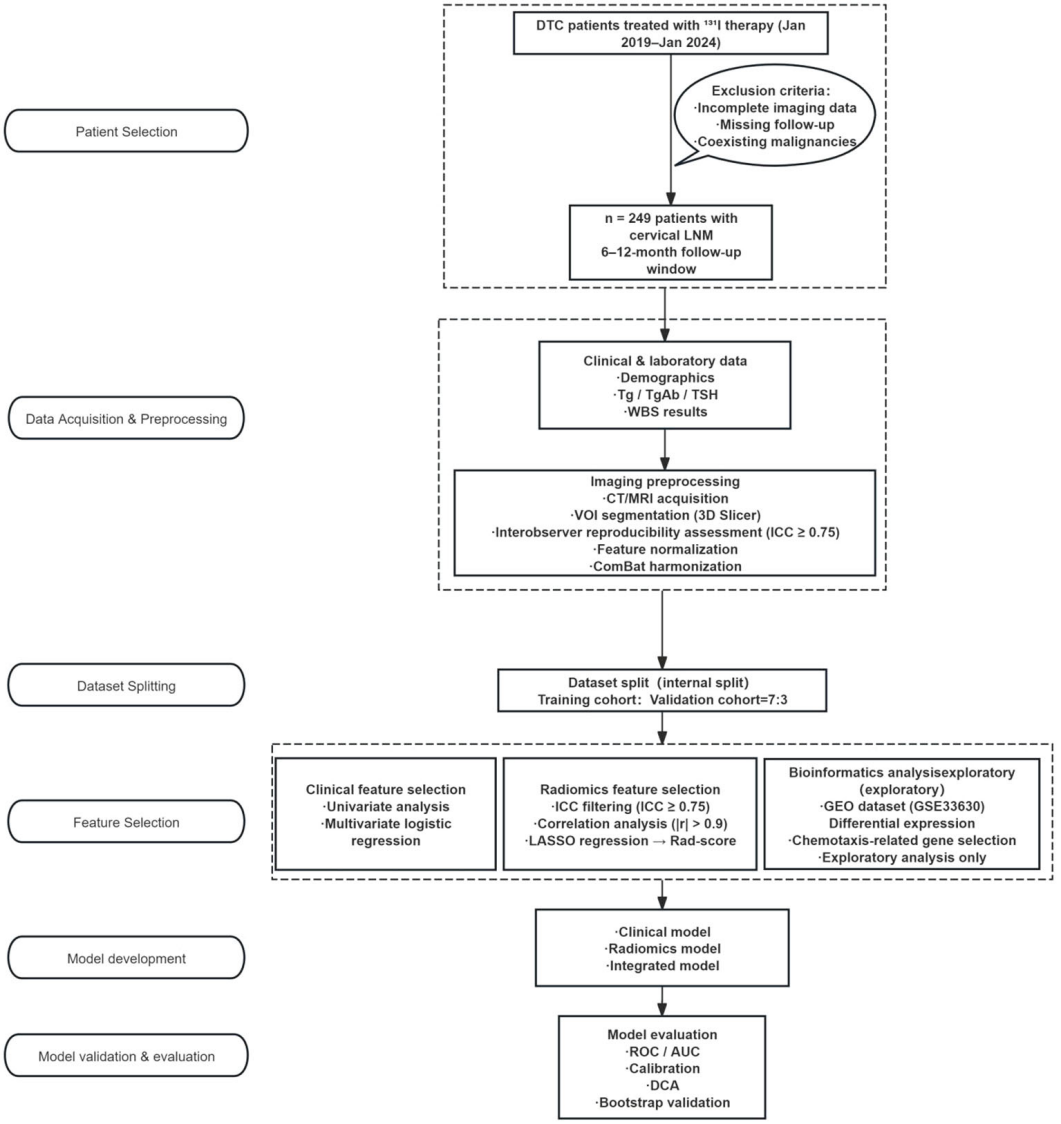
Workflow for treatment response evaluation in DTC patients after 131I therapy. Workflow of patient selection, data preprocessing, feature extraction, model development, and validation. The diagram illustrates patient inclusion and exclusion, imaging and clinical data preprocessing, dataset splitting, variable selection for clinical, radiomics, and bioinformatics features, and the construction and internal validation of predictive models. DTC, differentiated thyroid cancer; LNM, lymph node metastasis; Tg, thyroglobulin; TgAb, anti-thyroglobulin antibody; TSH, thyroid-stimulating hormone; WBS, whole-body scintigraphy; CT, computed tomography; MRI, magnetic resonance imaging; VOI, volume of interest; ICC, intraclass correlation coefficient; LASSO, least absolute shrinkage and selection operator; GEO, Gene Expression Omnibus; ROC, receiver operating characteristic; AUC, area under the curve; DCA, decision curve analysis.

**Figure 5:**
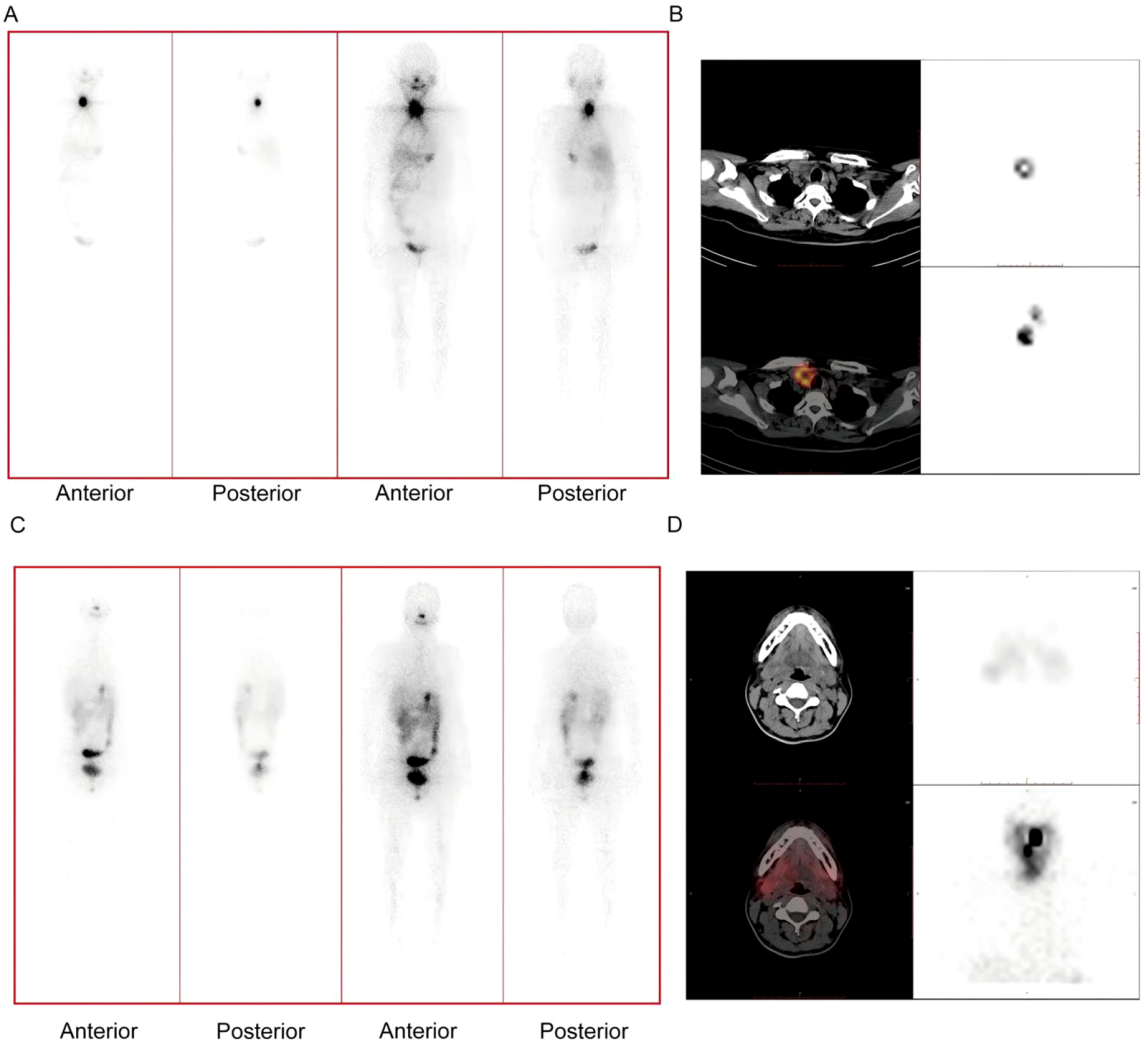
Representative 131I RX-WBS images. **(A, B)** Iodine-avid patients. **(A)** Planar anterior and posterior RX-WBS images demonstrate focal radioactive iodine uptake in the neck and thyroid bed, indicating sensitivity to ¹³¹I therapy. **(B)** Axial SPECT/CT images acquired at the neck/thoracic inlet level further localize the iodine-avid lesion, confirming lesion-associated tracer uptake. **(C, D)** Non–iodine-avid patients. **(C)** Planar anterior and posterior RX-WBS images show no lesion-related abnormal iodine uptake, with only physiological distribution in the salivary glands, gastrointestinal tract, and urinary bladder. **(D)** Axial SPECT/CT images obtained at a different cranio-caudal level (upper neck/oropharyngeal region) demonstrate the absence of focal iodine-avid lesions, showing only physiological tracer activity. Notably, panels **(B, D)** represent SPECT/CT images acquired at different anatomical levels and orientations from the planar whole-body scintigraphy images in panels **(A, C)**. These images are presented as representative examples of iodine-avid and non–iodine-avid patterns rather than for direct spatial correspondence. WBS, whole-body scintigraphy; RX, post-radioiodine therapy; SPECT: Single-photon emission computed tomography.

A total of 249 DTC patients with cervical LNM were enrolled. Based on the ATA response criteria, patients were divided into Excellent (n = 126) and non-Excellent (n = 123) groups (**Figure 4**). Post-therapeutic 131I whole-body scintigraphy (Rx-WBS) results are shown in **Figure 5**, and baseline characteristics are summarized in **Table 2**. All variables included in the analysis were complete, and no missing data were observed in the final study cohort.

**Table 2:** Comparison of baseline characteristics between the two patient groups.

Variable	Non-Excellent response group (n=123)	Excellent response group (n=126)	t/χ²/Z	*p* value
Age (years)	56.13 ± 12.04	52.13 ± 11.05	2.735	0.007
Sex: Female, n (%)	55 (44.72%)	75 (59.52%)	5.470	0.019
Pathology: PTC, n (%)	111 (90.24%)	112(88.89%)	0.122	0.727
Lymph node level: Central area VI, n (%)	59 (47.97%)	79 (62.7%)	5.467	0.019
ShortAxis baseline, mm	15.42 ± 3.84	10.97 ± 3.40	9.683	<0.001
Tg baseline, ng/mL	18.4 (15.2, 24.3)	12.15 (9.58, 15.03)	-9.02	<0.001
TgAb positivity: Positive	70 (56.91%)	69 (54.76%)	0.117	0.733
Baseline TSH suppression status, n (%)	51 (41.46%)	69 (54.76%)	4.409	0.036
FT3 baseline, pmol/L	4.18 ± 0.67	4.26 ± 0.71	-0.933	0.352
FT4 baseline, pmol/L	16.39 (14.92,18.11)	16.40 (14.29,18.39)	-0.294	0.769
WBS result:WBS positive	77 (62.6%)	108 (85.71%)	17.411	<0.001
LN count: >5 nodes	90 (73.17%)	61 (48.41%)	15.985	<0.001

Data are presented as mean ± SD or median [IQR]. Between-group differences were analyzed using t-test or Mann–Whitney U test for continuous variables and χ² test for categorical variables. Statistical significance was defined as *p* < 0.05. Baseline TSH suppression status: Adequate (0.1–0.5 mU/L). Tg, Thyroglobulin;TgAb, Anti-thyroglobulin antibody; TSH, Thyroid-stimulating hormone; FT3, Free triiodothyronine; FT4, Free thyroxine; WBS, Whole-body scintigraphy.

Compared with the non-Excellent response group, patients in the Excellent response group were younger (52.13 ± 11.05 vs. 56.13 ± 12.04 years, P = 0.007), had a higher female proportion (59.5% vs. 44.7%, P = 0.019), and showed a greater rate of central (level VI) LNM (62.7% vs. 48.0%, P = 0.019). Moreover, they had significantly smaller nodal short-axis diameters (10.97 ± 3.40 mm vs. 15.42 ± 3.84 mm, *p* < 0.001), lower baseline Tg levels (median 12.15 vs. 18.4 ng/mL, *p* < 0.001), and higher proportions of iodine-avid lesions (WBS positivity: 85.7% vs. 62.6%, *p* < 0.001).

### 3.5 Logistic regression analysis

**Figure 6:**
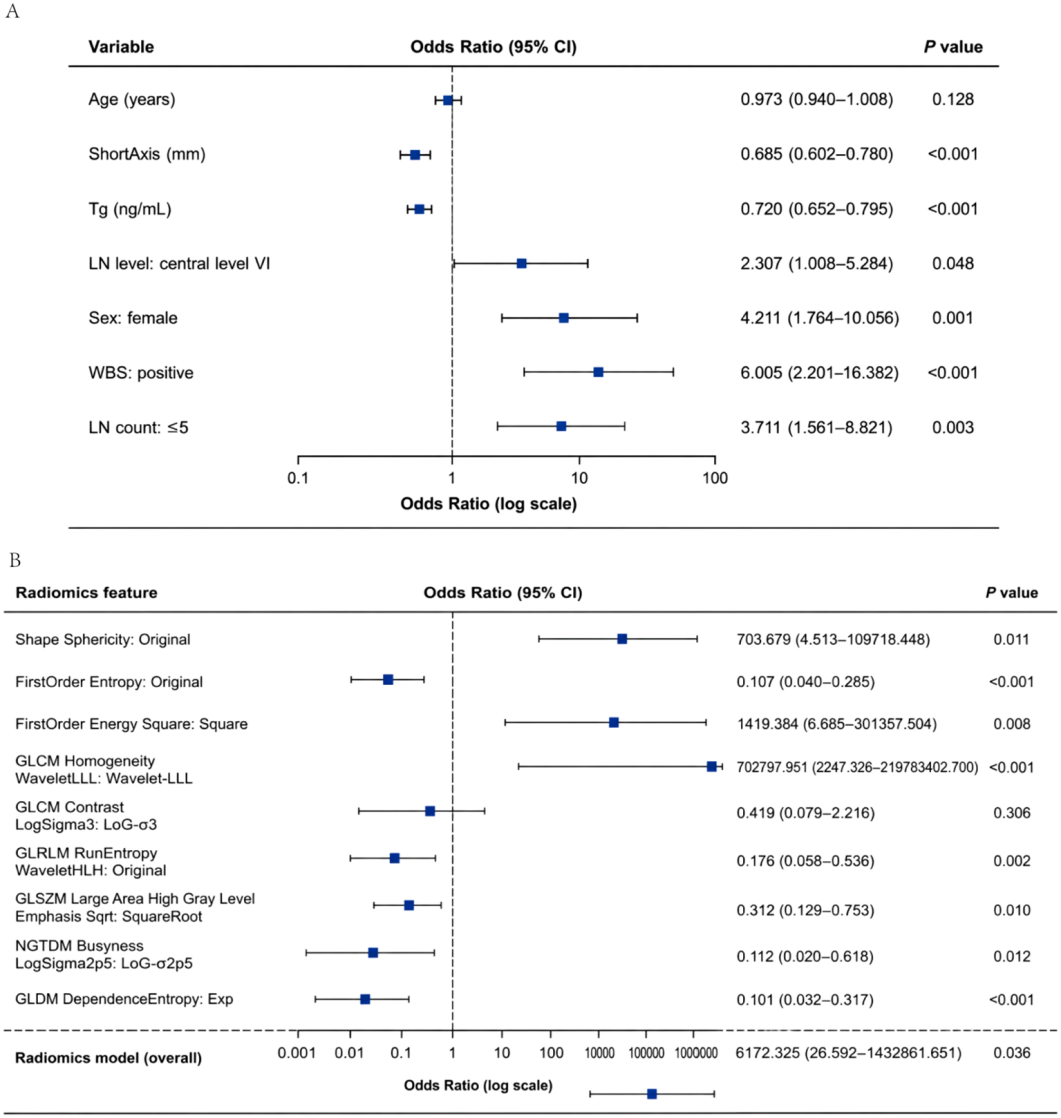
Forest plots of multivariable logistic regression analyses associated with treatment response to ^131I therapy. **(A)** Forest plot illustrating the independent clinical predictors associated with Excellent response to ^131I therapy. Variables included age, lymph node (LN) short-axis diameter, serum thyroglobulin (Tg) level, central LN level VI involvement, sex, whole-body scintigraphy (WBS) positivity, and LN count. The blue squares represent the odds ratios (ORs), and the horizontal lines indicate the corresponding 95% confidence intervals (CIs). The dashed vertical line represents the reference value (OR = 1). **(B)** Forest plot showing the multivariable logistic regression analysis of radiomics features associated with treatment response. The selected radiomics features included shape-based, first-order, gray-level co-occurrence matrix (GLCM), gray-level run-length matrix (GLRLM), gray-level size zone matrix (GLSZM), neighborhood gray-tone difference matrix (NGTDM), and gray-level dependence matrix (GLDM) parameters. ORs and corresponding 95% CIs are presented on a logarithmic scale. OR, odds ratio; CI, confidence interval; LN, lymph node; Tg, thyroglobulin; WBS, whole-body scintigraphy; GLCM, gray-level co-occurrence matrix; GLRLM, gray-level run-length matrix; GLSZM, gray-level size zone matrix; NGTDM, neighborhood gray-tone difference matrix; GLDM, gray-level dependence matrix.

Variables with significant differences in univariate analysis were entered into multivariate logistic regression (**Supplementary Tables 1**, **2**; **Figure 6A**). Short-axis diameter (ShortAxis_mm) (OR = 0.685, 95% CI: 0.602–0.780, *p* < 0.001) and baseline Tg (Tg_ng/mL) (OR = 0.720, 95% CI: 0.652–0.795, *p* < 0.001) were identified as negative predictors. Central level VI metastasis (LN_level = Central VI) (OR = 2.307, *p* = 0.048), female sex (OR = 4.211, *p* = 0.001), WBS positivity (OR = 6.005, *p* < 0.001), and ≤5 metastatic lymph nodes (OR = 3.711, *p* = 0.003) were independent protective factors. Age and TSH suppression status were not significant. In summary, patients with smaller nodes, lower Tg, central metastases, female sex, WBS positivity, and fewer metastatic nodes were more likely to achieve an Excellent response.

### 3.6 Radiomics feature analysis

Multivariate logistic regression (**Supplementary Table 3**, **Figure 6B**) identified nine radiomics features significantly associated with 131I treatment response. Elevated Shape Sphericity, FirstOrder Energy Square, and GLCM Homogeneity Wavelet-LLL correlated with Excellent responses, suggesting that lesions with more regular morphology and homogeneous texture were more 131I-sensitive. Conversely, increased FirstOrder Entropy, GLRLM RunEntropy Wavelet-HLH, GLSZM LargeAreaHighGrayLevelEmphasis Sqrt, NGTDM Busyness LogSigma2.5, and GLDM DependenceEntropy Exp were associated with poorer outcomes. Overall, radiomics revealed that compact and uniform structural phenotypes corresponded to better therapeutic responses.

### 3.7 Performance validation of the radiomics model

**Figure 7:**
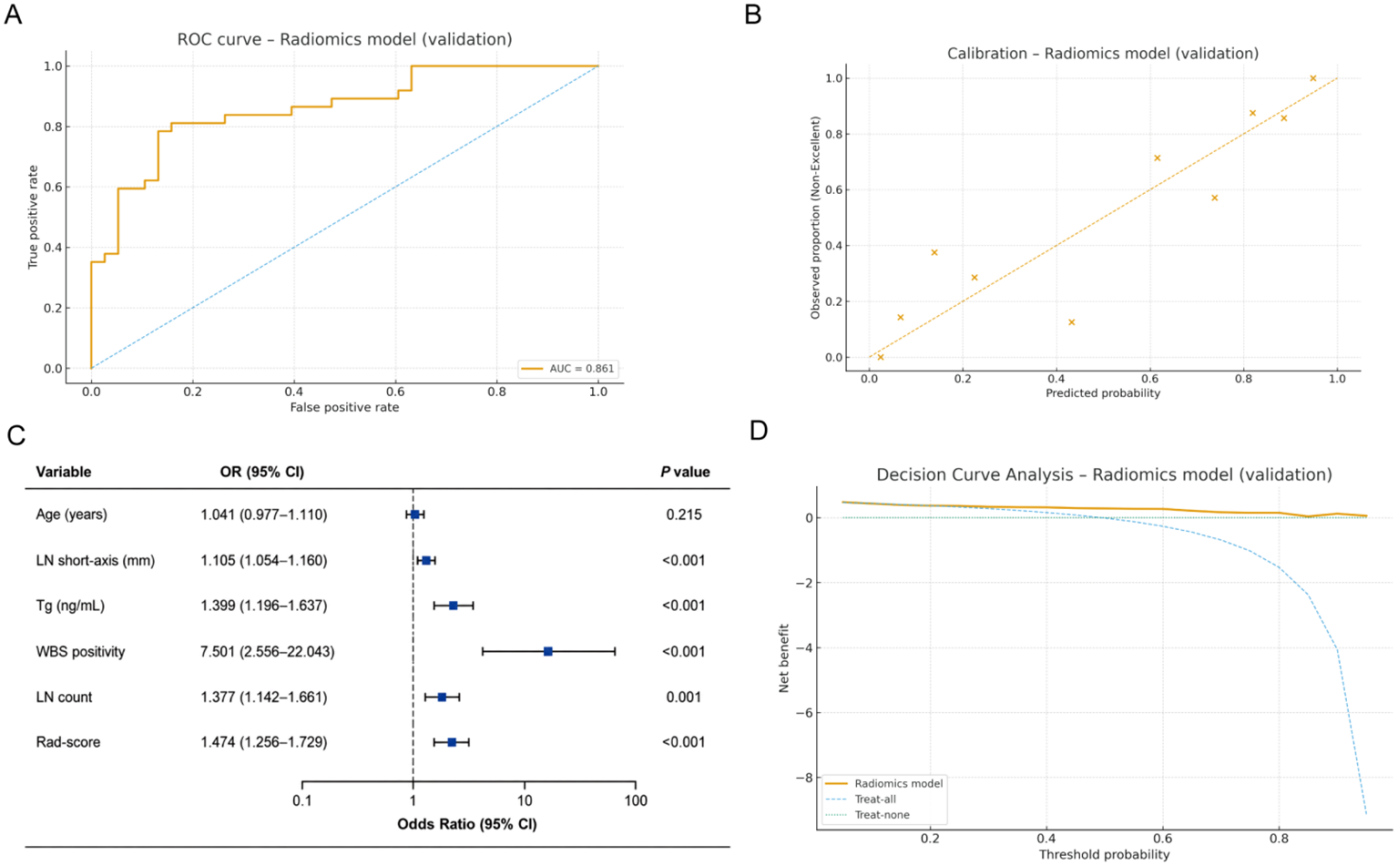
Performance of the radiomics model in the validation cohort. **(A)** ROC curve showing discriminative ability (AUC = 0.861); **(B)** Calibration curve demonstrating good agreement between predicted and observed outcomes; **(C)** Multivariable forest plot presenting ORs with 95% CIs for model features; **(D)** Decision curve analysis comparing the net clinical benefit of the radiomics model across a range of threshold probabilities. The radiomics model demonstrated higher net benefit than the treat-all and treat-none strategies across clinically relevant threshold probabilities. In panel D, the clinically relevant threshold probability range (10%–50%), within which the radiomics model demonstrates a higher net benefit than both the treat-all and treat-none strategies. Abbreviations: ROC, Receiver operating characteristic; AUC, Area under the curve; DCA, Decision curve analysis; CI, Confidence interval.

Patients were randomly divided into a training set and a validation set in a 70:30 ratio, and baseline characteristics were comparable between the two cohorts. Model performance in the training and validation sets is shown in **Table 3** and **Figure 7**. In the training cohort, AUC = 0.903 and accuracy = 0.822; in the validation cohort, AUC = 0.861 and accuracy = 0.787. The Hosmer–Lemeshow test indicated good calibration (P > 0.27). Decision curve analysis demonstrated that the integrated model provided a higher net benefit than the treat-all and treat-none strategies across a threshold probability range of approximately 10% to 50%, indicating potential clinical usefulness within this decision interval(**Figure 7D**). Additional stratified 5-fold cross-validation demonstrated stable predictive performance of the radiomics model, yielding a mean AUC of 0.877 ± 0.054 across the five folds. These findings support the robustness and reproducibility of the proposed model and suggest that the observed predictive performance was not solely dependent on a single train–validation split.

**Table 3:** Validation performance of the radiomics model.

Metric	Training cohort	Validation cohort
AUC	0.903	0.861
Accuracy	0.822	0.787
Sensitivity	0.814	0.811
Specificity	0.830	0.763
PPV	0.824	0.77
NPV	0.821	0.805
Hosmer–Lemeshow test	χ²(8)=4.355, *p* = 0.824	χ²(df=8) ≈ 7.1, *p* = 0.275

Hosmer–Lemeshow (HL) test was used to assess calibration; AUC indicates the area under the receiver operating characteristic curve. PPV and NPV were calculated based on the reported sensitivity and specificity, assuming an outcome prevalence of 49.4% (Non-Excellent response) consistent with the overall cohort. Abbreviations: AUC, Area under the curve;PPV, Positive predictive value; NPV, Negative predictive value; PPV, positive predictive value; NPV, negative predictive value; HL, Hosmer–Lemeshow test.

### 3.8 Correlation between radiomics risk phenotype and inflammatory index

**Figure 8:**
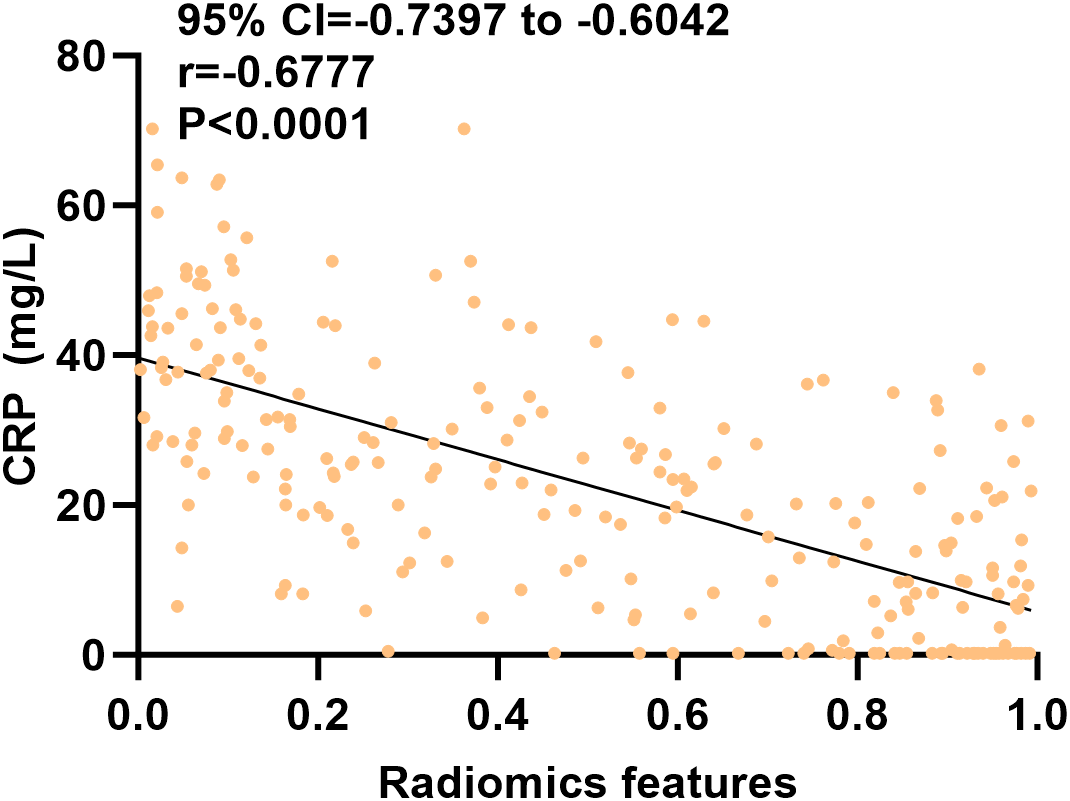
Correlation between radiomics risk phenotype and CRP level. CRP, C-reactive protein.

To explore whether the radiomics-derived risk phenotype was associated with systemic inflammatory status, we examined its correlation with CRP. We observed a significant negative correlation (r = −0.678, *p* < 0.001), with a moderate explained variance (R² = 0.46), suggesting that the imaging phenotype is partially associated with systemic inflammation but does not fully account for inflammatory variability. (**Figure 8**). Although CXCL8 demonstrated significant differential expression in the public transcriptomic dataset, no matched molecular profiling was available in the present clinical cohort. Therefore, the current findings should be interpreted as exploratory and hypothesis-generating rather than definitive radiogenomic validation.

### 3.9 Integrated predictive model and ROC validation

Combined clinical–radiomics model integrating key clinical variables and radiomics features was constructed to predict treatment response.

**Figure 9:**
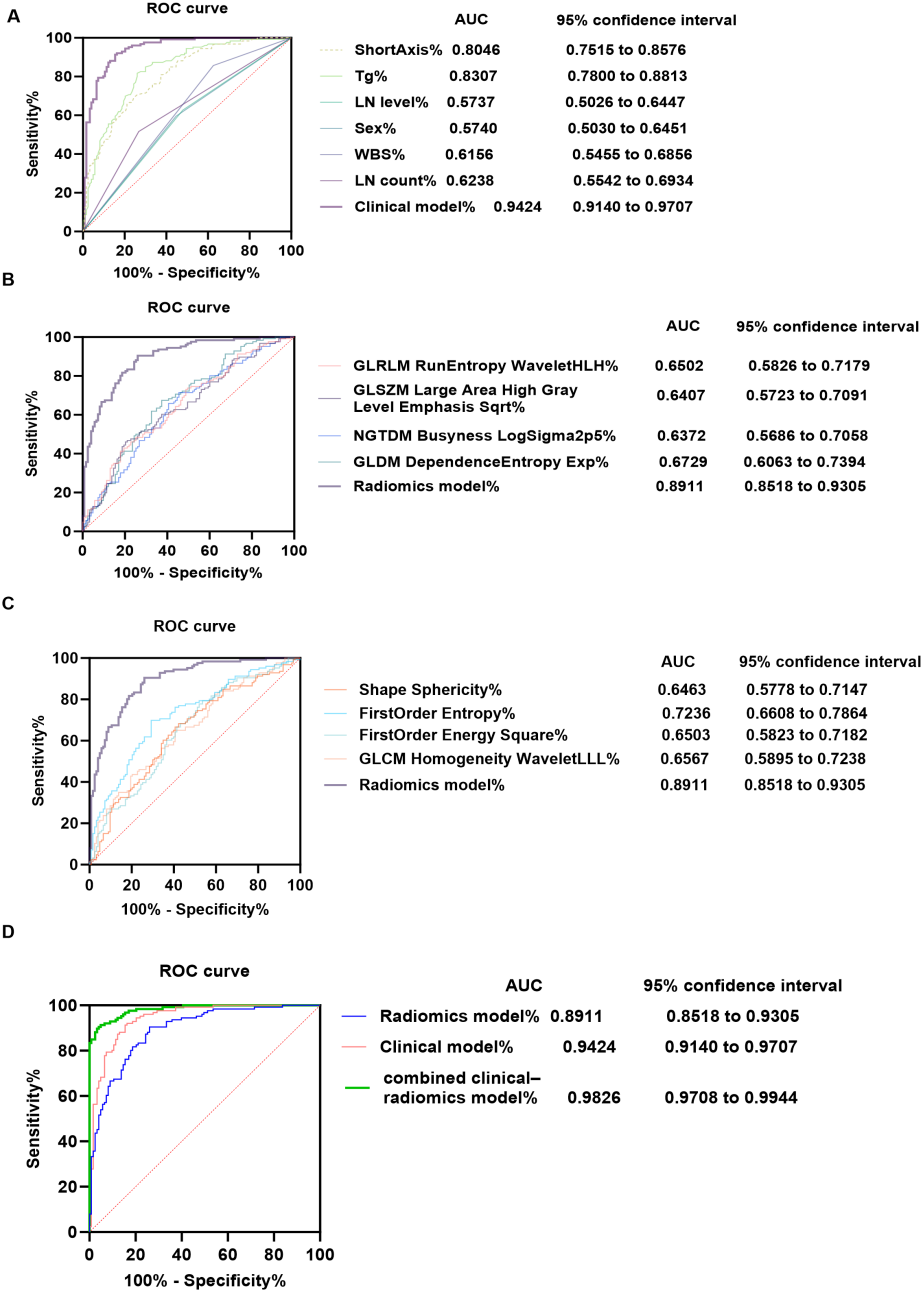
ROC analysis of clinical predictors, radiomics predictors, and the combined clinical–radiomics model. **(A)** ROC curve of the clinical model; **(B, C)** ROC curve of radiomics model; **(D)** ROC curve of the Combined clinical–radiomics model. AUC, Area under the curve; ROC, Receiver operating characteristic. The corresponding AUC values are displayed within the ROC curves to facilitate direct visual comparison of model performance.

As shown in **Figure 9A**, most individual clinical predictors demonstrated moderate discriminative ability, with Tg and lymph node short-axis diameter showing relatively better performance; however, their predictive power was still inferior to the integrated Clinical model (AUC = 0.9424, 95% CI: 0.9140–0.9707; sensitivity 91.27%; specificity 84.55%), as summarized in **Table 4**.

**Table 4:** ROC performance metrics of clinical, radiomics, and integrated models.

Variable	AUC	Youden	S.E.	95%CI	Sensitivity(%)	Specificity(%)
ShortAxis_mm	0.8046	0.4473	0.02708	0.7515 to 0.8576	65.87	78.86
Tg (ng/mL)	0.8307	0.5573	0.02584	0.7800 to 0.8813	81.75	73.98
LN level	0.5737	0.1473	0.03627	0.5026 to 0.6447	62.7	52.03
Sex	0.574	0.148	0.03626	0.5030 to 0.6451	59.52	55.28
WBS	0.6156	0.2311	0.03572	0.5455 to 0.6856	85.71	37.4
LN count	0.6238	0.2476	0.0355	0.5542 to 0.6934	51.59	73.17
Clinical model	0.9424	0.7582	0.01446	0.9140 to 0.9707	91.27	84.55
Shape Sphericity	0.6463	0.2602	0.03492	0.5778 to 0.7147	66.67	59.35
FirstOrder Entropy	0.7236	0.4057	0.03204	0.6608 to 0.7864	69.84	70.73
FirstOrder Energy Square	0.6503	0.2575	0.03467	0.5823 to 0.7182	77.78	47.97
GLCM Homogeneity WaveletLLL	0.6567	0.2443	0.03427	0.5895 to 0.7238	65.08	59.35
GLRLM RunEntropy WaveletHLH	0.6502	0.2424	0.03451	0.5826 to 0.7179	73.02	51.22
GLSZM Large Area High Gray Level Emphasis Sqrt	0.6407	0.257	0.0349	0.5723 to 0.7091	46.03	79.67
NGTDM Busyness LogSigma2p5	0.6372	0.259	0.035	0.5686 to 0.7058	71.43	54.47
GLDM DependenceEntropy Exp	0.6729	0.3006	0.03397	0.6063 to 0.7394	67.46	62.6
Radiomics model	0.8911	0.6446	0.0201	0.8518 to 0.9305	90.48	73.98
Combined clinical–radiomics model	0.9826	0.8643	0.006007	0.9708 to 0.9944	89.68	96.75

Model performance was evaluated using ROC analysis; AUC indicates discriminative ability. The integrated model combined clinical variables and radiomics features.

In **Figure 9B**, multiple discriminative radiomics features also showed good predictive value, whereas the Radiomics model consistently outperformed single radiomics features (AUC = 0.8911, 95% CI: 0.8518–0.9305; sensitivity 90.48%; specificity 73.98%), further confirming the complementary value of combined information (**Table 4**).

**Figure 10:**
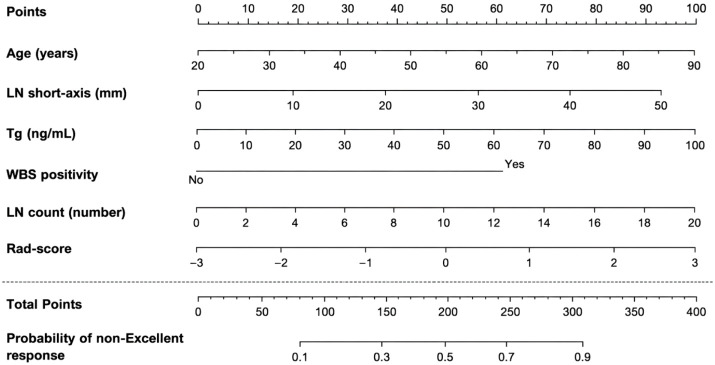
Simplified combined clinical–radiomics nomogram for predicting non-Excellent response to ^131I therapy in patients with differentiated thyroid cancer. The nomogram was constructed based on the combined clinical–radiomics model and incorporates six variables, including age, lymph node (LN) short-axis diameter, serum thyroglobulin (Tg) level, whole-body scintigraphy (WBS) positivity, LN count, and the integrated radiomics signature score (Rad-score). For each variable, a vertical line is drawn upward to the “Points” axis to determine the corresponding score. The total points are calculated by summing the scores of all variables and subsequently projected downward to estimate the probability of non-Excellent response to ^131I therapy. LN, lymph node; Tg, thyroglobulin; WBS, whole-body scintigraphy; Rad-score, radiomics signature score.

Finally, **Figure 9C** directly compared the radiomics-only model, other models, and the final comprehensive Combined clinical–radiomics model. The model achieved the best overall performance (AUC = 0.9826, 95% CI: 0.9708–0.9944; sensitivity 89.68%; specificity 96.75%), markedly outperforming the radiomics-only and clinical-only strategies (**Table 4**). The integrated model demonstrated excellent discrimination in the validation cohort (AUC = 0.9826). Nevertheless, given the retrospective single-center design and the relatively limited cohort size, some degree of optimism in model performance cannot be completely excluded despite bootstrap-based internal validation. Although the model demonstrated strong discriminative performance in the validation cohort, the results should still be interpreted cautiously given the single-center retrospective design and the absence of external validation. A simplified combined clinical–radiomics nomogram was constructed based on the final integrated model to estimate the probability of Non-Excellent response to ^131I therapy (**Figure 10**).

## 4 Discussion

### 4.1 Main findings and clinical implications

In this study, based on 249 postoperative patients with cervical metastatic DTC, we systematically analyzed the multidimensional factors influencing the therapeutic response to radioactive 131I therapy. The results demonstrated that the short-axis diameter of lymph nodes, serum Tg level, sex, metastatic distribution level, WBS findings, and number of metastatic lesions were the key determinants of treatment efficacy. Patients with smaller short-axis diameter, lower Tg levels, female sex, central compartment metastases, and iodine-avid lesions were more likely to achieve an excellent response, suggesting that the volume and functional status of metastatic lesions play central roles in determining 131I treatment sensitivity (29). This finding is consistent with previous observations that “micrometastases are more sensitive to radioactive iodine uptake” (30), further confirming the negative correlation between metastatic burden and treatment response.

The introduction of a radiomics-based model significantly improved the predictive performance of therapeutic response. Among radiomic features, sphericity and homogeneity were identified as independent protective factors, indicating that lesions with more regular structures and uniform signal intensities are associated with greater treatment sensitivity (31). In contrast, increased entropy and complex texture features indicated greater tissue heterogeneity, reflecting higher rates of cell proliferation and necrosis, which impair iodine uptake (32). The final integrated model achieved an AUC of 0.9826, demonstrating excellent discriminative power and highlighting the clinical translational potential of multimodal models for predicting 131I therapy response. Recent studies applying radiomics and machine learning in thyroid cancer management have reported AUC values typically ranging from approximately 0.80 to 0.92 in predicting treatment response or recurrence risk, depending on cohort characteristics and modeling strategies. Compared with these state-of-the-art approaches, our integrated clinical–radiomics framework achieved comparable or superior discrimination, while also incorporating biologically interpretable molecular information, which may provide incremental clinical insight. Nevertheless, methodological heterogeneity across studies limits direct head-to-head comparison, and further multi-center benchmarking is warranted.

### 4.2 Mechanistic interpretation: the role of the chemokine–inflammation axis

Bioinformatics analysis revealed significant activation of chemokine-related pathways (chemokine-mediated signaling and leukocyte chemotaxis) in DTC, with CXCL8 consistently enriched across multiple pathways (33–35) and verified by the Human Protein Atlas (HPA) database to be highly expressed in tumor tissues. CXCL8, as a key mediator of the immune-inflammatory microenvironment, promotes tumor cell migration, enhances angiogenesis, and disrupts sodium/iodide symporter (NIS) function (36), thereby reducing iodine uptake efficiency (37). In our study, the radiomic risk phenotype was significantly negatively correlated with C-reactive protein (CRP) levels, suggesting that radiomic heterogeneity may reflect inflammation-driven microenvironmental activation consistent with chemokine signaling upregulation.

### 4.3 Comparison with previous studies

Previous studies on radioactive iodine therapy in DTC have mainly focused on clinicopathological or molecular factors, lacking cross-level integration. Some reports identified Tg, TgAb, and WBS iodine uptake status as important predictors of treatment efficacy (38, 39). Texture parameter analyses from CT imaging have also shown that higher entropy values are associated with increased risk of RAIR disease (40, 41). Building upon these findings, our study introduced a “bioinformatics–radiomics integration” strategy, combining imaging-derived structural characteristics with molecular chemotactic mechanisms, thereby bridging the association between imaging phenotype and functional response. This approach substantially enhances both predictive accuracy and biological interpretability.

### 4.4 Clinical applications and future prospects

The integrated model (Alliance) enables noninvasive prediction of therapeutic outcomes during postoperative follow-up through conventional CT or magnetic resonance imaging (MRI) combined with basic serological indices. This can assist in individualized decision-making regarding 131I dosage and follow-up frequency, avoiding ineffective or excessive treatment. Moreover, the persistently high expression of CXCL8 and related chemokine axes suggests their potential as therapeutic targets, providing a molecular intervention strategy for RAIR patients. Importantly, the present bioinformatics findings were derived from external public datasets rather than matched molecular sequencing within the included clinical cohort. Therefore, the observed association between radiomics features and CXCL8-related biological pathways should be interpreted cautiously as biologically supportive rather than constituting direct radiogenomic validation.

From a clinical perspective, individualized radioiodine dosimetry has increasingly been recognized as an important factor influencing therapeutic response, as absorbed dose heterogeneity may partly explain differences in outcomes among patients receiving seemingly similar treatment protocols. For patients who fail to respond adequately or develop RAIR disease, alternative therapeutic strategies such as TKI-targeted therapy, selective internal radiation approaches, and careful surveillance are routinely considered in clinical practice. Therefore, predictive tools such as our model may assist in early identification of potentially poor responders, facilitating more timely risk stratification and individualized therapeutic planning.

Future studies integrating radiomic–immunologic analyses and multicenter external validation are warranted to further verify the model’s robustness and clinical applicability. The threshold probability range of 10–50% reflects clinically plausible decision thresholds for identifying patients at risk of Non-Excellent response, where the potential benefits of risk-adapted management may outweigh unnecessary interventions. Outside this range, the net clinical benefit of prediction-guided decisions is limited. It should be emphasized that the sharp decline of the “treat-all” curve after approximately the 10% threshold does not indicate a flaw of the model; rather, it highlights the inherent clinical limitation of indiscriminate treatment. When the threshold probability enters the medium-to-high range, treating all patients inevitably leads to substantial overtreatment, and the harm caused by unnecessary interventions outweighs the potential benefit, resulting in markedly negative net benefit. In contrast, the radiomics model remains close to or above the zero net-benefit line across this range, indicating that the model effectively reduces unnecessary treatment and preserves meaningful clinical value. Therefore, our interpretation primarily focused on the 10%–50% threshold range, where the treat-all strategy still lies within a clinically reasonable, non-negative region. Within this interval, the radiomics model consistently provides greater net benefit than both “treat-all” and “treat-none,” supporting its practical usefulness in real-world decision-making.

Although the proposed model demonstrates promising predictive ability, its current role should be regarded as an adjunctive decision-support tool rather than a replacement for clinical judgment. The findings should be interpreted cautiously, as external validation and prospective testing are still required before routine clinical deployment.

### 4.5 Limitations and future directions

This was a single-center retrospective study; although the sample size was adequate, potential selection bias cannot be excluded. Imaging data were acquired from multiple devices, and although ComBat harmonization was applied, residual batch effects may persist. Furthermore, molecular findings such as CXCL8 expression were derived from public databases and lacked matched histopathological validation from patient tissues. Several limitations should be considered when interpreting the imaging–inflammation linkage. First, the association between radiomics phenotypes and the chemotaxis-related signal (including CXCL8) is correlational, derived from public transcriptomic data rather than paired molecular measurements in our cohort; therefore, causal inference cannot be made. Second, CRP is a non-specific systemic inflammatory marker and may be influenced by comorbid infections, postoperative status, and other clinical factors, which limits its ability to serve as a direct surrogate of tumor-local chemokine activity. Third, the correlation in Figure shows a moderate explained variance (R² = 0.46), indicating that radiomics heterogeneity captures only part of inflammation-related variation, and additional biological processes and clinical covariates likely contribute. Future studies incorporating matched tissue immunohistochemistry or circulating cytokine profiling (e.g., IL-8/CXCL8) and prospective multicenter validation are warranted to strengthen mechanistic interpretation. Subgroup analysis was considered; however, due to the limited sample size of individual subcategories, performing stratified comparisons may introduce statistical instability. Furthermore, the bioinformatics analysis was based on external public datasets rather than biological material from the patients included in this radiomics cohort. Therefore, the radiomics–chemokine associations should be interpreted as hypothesis-generating and exploratory. Future studies incorporating matched molecular profiling, serum biomarkers, or tissue-based assays are required to achieve true radiogenomic validation. Therefore, to avoid unreliable interpretation, subgroup analyses were not presented, which is acknowledged as a limitation of this study. Although our sample size met contemporary recommendations for prediction model development (including both events-per-variable considerations and formal calculations using the pmsampsize framework), the present study remains a single-center cohort with internal validation only. Therefore, some degree of optimism in the apparent model performance cannot be excluded, and larger multicenter cohorts with external validation are needed to confirm the generalizability of our findings. The current findings should be considered preliminary and hypothesis-supportive, and future multicenter prospective validation is necessary before routine clinical implementation. In addition, although CXCL8-related transcriptomic findings provided potential biological support for the radiomics signatures, external validation using independent transcriptomic cohorts, such as TCGA-THCA or additional GEO datasets, as well as prospective molecular validation in matched clinical samples, is still required to establish definitive radiogenomic associations. Although the integrated model achieved excellent discriminative performance, some degree of optimism may still exist because of the retrospective single-center design. Nevertheless, the additional 5-fold cross-validation analysis demonstrated stable predictive performance (mean AUC = 0.877 ± 0.054), supporting the robustness of the proposed radiomics framework. Future multicenter external validation remains necessary to confirm its generalizability. An additional limitation is that ComBat harmonization was performed during the original radiomics preprocessing workflow, and scanner-specific harmonization parameters were not available for retrospective re-estimation within each training fold. Therefore, a residual risk of harmonization-related information leakage cannot be completely excluded. Future multicenter studies should incorporate harmonization procedures entirely within the training pipeline and evaluate their impact through external validation.

## 5 Conclusion

This study proposes a multimodal model integrating chemokine-axis bioinformatics with radiomics to predict 131I response in cervical metastatic DTC, highlighting the potential role of inflammatory–chemotactic signaling in treatment sensitivity and supporting more individualized therapy. The model shows promising predictive potential, but further prospective and multi-center evaluation is required before routine clinical implementation.

## Data Availability

The original contributions presented in the study are included in the article/**Supplementary Material**. Further inquiries can be directed to the corresponding author.
