# Supplementary material for: Cervical lymph node response to 131I therapy in differentiated thyroid cancer using radiomics and clinical features
Source: Front Oncol. 2026 Jun 23;16:1860423. doi: 10.3389/fonc.2026.1860423 (PMC13337463; doi:10.3389/fonc.2026.1860423)
Supplement: Supplementary file 1 [file Table1.docx]

**Table S1. Variable coding for logistic regression analysis**

| Variables | Coding |
| --- | --- |
| Grouping | Non-Excellent response=1, Excellent response=2 |
| Sex | male=1, female=2 |
| Age, years | Continuous variable |
| ShortAxis, mm | Continuous variable |
| Tg, ng/mL | Continuous variable |
| LN level | Lateral neck =1, Central compartment (level VI)=2 |
| TSH suppression | Poor suppression (TSH <0.1 or >0.5 mU/L)=1,  Adequate suppression (0.1–0.5)=2 |
| WBS | Non–iodine-avid(WBS-)=1, Iodine-avid(WBS+)=2 |
| LN count | >5 metastatic nodes =1, ≤5 metastatic nodes=2 |

Abbreviations: Tg, thyroglobulin; TgAb, anti-thyroglobulin antibody; TSH, thyroid-stimulating hormone; WBS, whole-body scintigraphy; LN, Lymph node.

**Table S2. Results of logistic regression analysis (predicting Excellent response)**

| Variable | β | SE | Wald | *p* value | OR (95% CI) |
| --- | --- | --- | --- | --- | --- |
| Age ,years | -0.027 | 0.018 | 2.319 | 0.128 | 0.973(0.94-1.008) |
| ShortAxis, mm | -0.378 | 0.066 | 32.887 | <0.001 | 0.685(0.602-0.78) |
| Tg, ng/mL | -0.328 | 0.05 | 42.657 | <0.001 | 0.72(0.652-0.795) |
| LN level: central level VI | 0.836 | 0.423 | 3.913 | 0.048 | 2.307(1.008-5.284) |
| Sex: female | 1.438 | 0.444 | 10.483 | 0.001 | 4.211(1.764-10.056) |
| TSH suppression:adequate | 0.375 | 0.423 | 0.782 | 0.376 | 1.454(0.634-3.335) |
| WBS: positive | 1.793 | 0.512 | 12.257 | <0.001 | 6.005(2.201-16.382) |
| LN count: ≤5 | 1.311 | 0.442 | 8.808 | 0.003 | 3.711(1.561-8.821) |
| Clinical model | 8.382 | 1.609 | 27.132 | <0.001 | 4366.945 |

Continuous variables are on the original scale. OR > 1 indicates a protective factor; OR < 1 indicates a risk factor. Abbreviations: OR, odds ratio; CI, confidence interval; SE, standard error; Tg, thyroglobulin; TSH, thyroid-stimulating hormone, LN, Lymph node; WBS, whole-body scintigraphy.

**Table S3. Multivariate regression analysis of radiomics features associated with treatment response**

| Radiomics feature | β | SE | Wald | *p* value | OR (95% CI) |
| --- | --- | --- | --- | --- | --- |
| Shape Sphericity:Original | 6.556 | 2.576 | 6.477 | 0.011 | 703.679  (4.513-109718.448) |
| FirstOrder Entropy:Original | -2.236 | 0.5 | 19.997 | <0.001 | 0.107(0.04-0.285) |
| FirstOrder Energy Square:Square | 7.258 | 2.734 | 7.049 | 0.008 | 1419.384  (6.685-301357.504) |
| GLCM Homogeneity WaveletLLL:Wavelet-LLL | 13.463 | 2.931 | 21.093 | <0.001 | 702797.951  (2247.326-219783402.7) |
| GLCM Contrast LogSigma3:LoG-σ3 | -0.869 | 0.85 | 1.047 | 0.306 | 0.419(0.079-2.216) |
| GLRLM RunEntropy WaveletHLH:Original | -1.736 | 0.568 | 9.343 | 0.002 | 0.176(0.058-0.536) |
| GLSZM Large Area High Gray Level Emphasis Sqrt:SquareRoot | -1.164 | 0.449 | 6.711 | 0.01 | 0.312(0.129-0.753) |
| NGTDM Busyness LogSigma2p5:LoG-σ25 | -2.19 | 0.872 | 6.306 | 0.012 | 0.112(0.02-0.618) |
| GLDM DependenceEntropy Exp | -2.295 | 0.585 | 15.383 | <0.001 | 0.101(0.032-0.317) |
| Radiomics model | 8.728 | 4.157 | 4.407 | 0.036 | 6172.325 |

*Abbreviations*: GLCM, gray-level co-occurrence matrix; GLRLM, gray-level run-length matrix; GLSZM, gray-level size zone matrix; NGTDM, neighborhood gray-tone difference matrix; GLDM, gray-level dependence matrix. *Note*: “Protective” indicates positive correlation with Excellent response; “Risk” indicates association with Non-Excellent response.
